# ^Utility of plasma GFAP in differentiating neurodegenerative from non‐neurodegenerative cognitive impairment: A real‐world clinical experience^

**DOI:** 10.1002/alz70856_107570

**Published:** 2026-01-09

**Authors:** Marian Vives Crook, Eduard Bargay Pizarro, Guillermo Amer Ferrer, Margalida Sastre Mesquida, Susana Tarongi Sanchez, Ana García Martin, Lara Nuñez Santos, María Santés Bertó, Daniel Morell García

**Affiliations:** ^1^ Son Espases University Hospital, Palma, Spain

## Abstract

**Background:**

Fluid biomarkers for Alzheimer's Disease (AD) have demonstrated strong diagnostic performance in clinical practice. However, distinguishing non‐AD neurodegenerative from non‐neurodegenerative cognitive impairment remains challenging. This study evaluates the utility of plasma glial fibrillary acidic protein (pGFAP) in addressing this diagnostic dilemma.

**Methods:**

We recruited 160 patients from our memory clinic (July 2022‐May 2024) and collected clinical, neuropsychological, and neuroimaging data, along with core AD cerebrospinal fluid (CSF) biomarkers. Plasma GFAP levels were concurrently measured using CMIA assay on the Alinity i series platform (limit of quantification: 3.2 pg/mL; intra‐assay CV <5%). Patients with immune‐mediated dementia were excluded. After excluding AD patients based on the Aß42/40 or ptau181/Aß42 indexes, 46 non‐AD patients remained. Neurologists, blinded to pGFAP results, classified participants either as neurodegenerative or non‐neurodegenerative based on clinical and imaging data. Group comparisons were performed using Wilcoxon rank‐sum tests, and robust regression models were used to adjust for confounding variables (age and sex).

**Results:**

Among the 46 non‐AD patients (69.9% female; median age 71.3 years [IQR: 63.1–76.1], median MMSE score was 27 [IQR: 22–28]), 17 were classified as neurodegenerative (FTD, LBD, LATE) and 29 as non‐neurodegenerative, including vascular cognitive impairment. Patients in the neurodegenerative group were older (median age 75 vs. 66 years, *p* =  0.004), with no significant differences in sex distribution. Median pGFAP levels were significantly higher in the neurodegenerative group (38.8 pg/mL [IQR: 27.8–45.4]) versus the non‐neurodegenerative group (21.7 pg/mL [IQR: 16.2–33.2], *p* =  0.001). ROC analysis yielded an AUC of 0.79 (95% CI: 0.66–0.92) for pGFAP, with a threshold of 35.6 pg/mL achieving 82.8% sensitivity and 64.7% specificity. Positive and negative predictive values were 62.9% and 83.8%, respectively. Robust regression analysis confirmed pGFAP's independent association with neurodegenerative diagnoses (*p* = 0.015), adjusting for age and sex.

**Conclusions:**

These findings support the utility of plasma GFAP, measured using a CMIA‐based assay, in differentiating neurodegenerative from non‐neurodegenerative cognitive impairments. Our real‐world cohort analysis underscores the potential of pGFAP for broader clinical application beyond AD.

